# Heavy Metal Toxicity in Armed Conflicts Potentiates AMR in *A. baumannii* by Selecting for Antibiotic and Heavy Metal Co-resistance Mechanisms

**DOI:** 10.3389/fmicb.2020.00068

**Published:** 2020-02-03

**Authors:** Wael Bazzi, Antoine G. Abou Fayad, Aya Nasser, Louis-Patrick Haraoui, Omar Dewachi, Ghassan Abou-Sitta, Vinh-Kim Nguyen, Aula Abara, Nabil Karah, Hannah Landecker, Charles Knapp, Megan M. McEvoy, Muhammad H. Zaman, Paul G. Higgins, Ghassan M. Matar

**Affiliations:** ^1^Department of Experimental Pathology, Immunology and Microbiology, Faculty of Medicine, American University of Beirut, Beirut, Lebanon; ^2^Center for Infectious Diseases Research, American University of Beirut, Beirut, Lebanon; ^3^World Health Organisation (WHO) Collaborating Center for Reference and Research on Bacterial Pathogens, Beirut, Lebanon; ^4^Department of Microbiology and Infectious Diseases, University of Sherbrooke, Sherbrooke, QC, Canada; ^5^Rutgers, The State University of New Jersey, Newark, NJ, United States; ^6^Faculty of Medicine, American University of Beirut, Beirut, Lebanon; ^7^The Graduate Institute of International and Developmental Studies, Geneva, Switzerland; ^8^Department of Infection, Imperial College London, London, United Kingdom; ^9^Department of Molecular Biology, Umea University, Umea, Sweden; ^10^Department of Sociology and Institute for Society and Genetics, University of California, Los Angeles, Los Angeles, CA, United States; ^11^Civil and Environmental Engineering, University of Strathclyde, Glasgow, United Kingdom; ^12^Institute for Society and Genetics, University of California, Los Angeles, Los Angeles, CA, United States; ^13^Department of Biomedical Engineering, Boston University, Boston, MA, United States; ^14^Institute for Medical Microbiology, Immunology and Hygiene, University of Cologne, Cologne, Germany; ^15^German Centre for Infection Research (DZIF), Partner Site Bonn-Cologne, Cologne, Germany

**Keywords:** *Acinetobacter baumannii*, bacteria, heavy metals, heavy metal tolerance, antimicrobial resistance, conflict, weapons

## Abstract

*Acinetobacter baumannii* has become increasingly resistant to leading antimicrobial agents since the 1970s. Increased resistance appears linked to armed conflicts, notably since widespread media stories amplified clinical reports in the wake of the American invasion of Iraq in 2003. Antimicrobial resistance is usually assumed to arise through selection pressure exerted by antimicrobial treatment, particularly where treatment is inadequate, as in the case of low dosing, substandard antimicrobial agents, or shortened treatment course. Recently attention has focused on an emerging pathogen, multi-drug resistant *A. baumannii* (MDRAb). MDRAb gained media attention after being identified in American soldiers returning from Iraq and treated in US military facilities, where it was termed “Iraqibacter.” However, MDRAb is strongly associated in the literature with war injuries that are heavily contaminated by both environmental debris and shrapnel from weapons. Both may harbor substantial amounts of toxic heavy metals. Interestingly, heavy metals are known to also select for antimicrobial resistance. In this review we highlight the potential causes of antimicrobial resistance by heavy metals, with a focus on its emergence in *A. baumanni* in war zones.

## Introduction

### Overview on Wars and Antimicrobial Resistance (AMR)

Over the past decades, *Acinetobacter baumannii* has emerged as a major driver of hospital-acquired Multi-Drug Resistant (MDR) infections (Manchanda et al., 2010; Williams et al., 2016; Mancilla-Rojano et al., 2019). In the decades following the Second World War, *A. baumannii* became one of the most prevalent pathogens during wars in Lebanon, Afghanistan, and Iraq, causing multiple outbreaks of MDR infections among combat casualties (Tong, 1972; CDC, 2004; Scott et al., 2007; Dallo and Weitao, 2010). According to The Centers for Disease Control and Prevention (CDC), *A. baumannii* was the single most isolated Gram-negative bacterium from war wounds during the recent wars in Iraq and Afghanistan and the number one causative agent of bloodstream infections among the US soldiers (CDC, 2004; Fournier et al., 2006). Moreover, the emergence of MDR, Extensively-Drug Resistant (XDR), and Pan-Drug Resistant (PDR) *A. baumannii* coincided with specific worldwide tension points such as the Lebanese Civil War (1975–1990) (Matar et al., 1992), Iraq-Iran war (1980–1988), Afghanistan war (2001–2014), Iraq war (2003–2011), and recently the Syrian war (2011–Present) (Tong, 1972; CDC, 2004; Scott et al., 2007). Warfare is associated with significant heavy metal contamination of the environment, due to destruction of built infrastructure and consequent release of HM and direct contamination from exploded ordnance and leakage from unexploded ordnance. HM resistance in bacteria is associated with antimicrobial resistance. In this review we investigate how heavy metal resistance can lead to antimicrobial resistance with a view to illuminating the emergence *A. baumannii*’s increased resistance in war regions.

### Living Systems and Heavy Metals

Heavy metals are a group of non-biodegradable metals and semi-metals (Metalloids) with high atomic weight and a density greater than 5 g/cm^3^. They have high electrical conductivity, malleability, metallic luster, and are capable of transferring electrons to form Cations (Jarup, 2003; Appenroth, 2010; Tchounwou et al., 2012). The major list of heavy metals includes: Cobalt (Co), Copper (Cu), Chromium (Cr), Zinc (Zn), Lead (Pb), Mercury (Hg), Arsenic (As), Cadmium (Cd), Nickel (Ni), Antimony (Sb), Boron (B), Barium (Ba), Silver (Ag), and Tungsten (W) (ATSDR, 2011). Essential heavy metals are required in many biological processes at low concentrations, where they serve as cofactors for metalloenzymes. For example, Cu serves as an integral cofactor for cytochrome c oxidase and superoxide dismutase required in mitochondrial electron transport and oxidative stress (Osredkar and Šuštar, 2011). Zn plays role in DNA and RNA polymerases catalytic functions (Markov et al., 1999). Ni and Cr are essential for the activity of urease and cytochrome P450 enzymes (Seiler and Berendonk, 2012). On the other hand, non-essential heavy metals such as Hg, Pb, Cd, and As have no known biological functions and are toxic even at low concentrations (ATSDR, 2011; Singh R. et al., 2011; Tchounwou et al., 2012).

Sources of heavy metals include water, soil, and rocks. Furthermore, they are present in the Earth’s crust and date back to 4.5 billion years ago (Fru et al., 2016). The ecosystem is continuously flooded with high amounts of heavy metals due to volcanic eruptions, soil and rock erosion, industrial operations such as petroleum combustion and coal burning, in addition to agricultural activities that include pesticides and fungicides preparation (Seiler and Berendonk, 2012; Tchounwou et al., 2012; Jaroslawiecka and Piotrowska-Seget, 2014; Pal et al., 2017). Furthermore, heavy metals have been used in medical treatments as antimicrobial and anti-parasitic agents to treat skin infections and leishmaniasis respectively; anti-inflammatory compounds to treat itchiness, and in chemotherapy to treat cancer patients (Rizzotto, 2012; Ndagi et al., 2017; Pal et al., 2017).

### Heavy Metals-Mediated Bacterial Toxicity

Essential and non-essential heavy metals become toxic when exceeding specific concentrations that vary between different metals (Appenroth, 2010). In bacteria, toxic levels of heavy metals can accumulate in cells, altering cellular processes, inducing structural modifications, and ultimately leading to heavy metals-mediated damage (Rouch et al., 1995). Mechanisms of heavy metals toxicity include the production of Reactive Oxygen Species (ROS) that destroy essential biomolecules and sub-cellular organelles. When in excess, Pb, Cd, Cu, As, Ag, and Zn can induce oxidative damage in bacterial cells which leads to the production of free radicals that cause DNA damage and destabilize membranous integrity through lipid peroxidation (Szivák et al., 2009; Jaroslawiecka and Piotrowska-Seget, 2014; Williams et al., 2016). In addition, heavy metal ions can form complexes with thiol (R-SH)-containing enzymes to alter their functions. For example, Hg^2+^, Ag^1+^, and Cd^2+^ can form covalent bonds with sulfhydryl functional groups (R-SH) present in enzymatic active sites thereby inducing structural conformational changes, and thus blocking their function (Rouch et al., 1995). Furthermore, heavy metals can act as competitive inhibitors leading to the displacement of essential ions from their target sites (Ianeva, 2009; Jan et al., 2015).

### Heavy Metals Resistance in Bacteria

Studies have shown that the presence of heavy metal resistance determinants is ubiquitous in almost all bacterial species (Silver and Phung le, 2005). For example, Farias et al. (2015), identified 35 bacterial strains from 8 different species harboring multi-metal resistant phenotypes from deep-sea hydrothermal vents. Moreover, *A. baumannii* isolated from agricultural soil and sediments, fuel-contaminated soil, and sewage water, have shown to exhibit resistance to various metals such as Hg, Ag, and As (Dhakephalkar and Chopade, 1994; Lima de Silva et al., 2012; Farias et al., 2015; Kim et al., 2015; El-Sayed, 2016; Huang et al., 2017). To date, six proposed mechanisms of heavy metal resistance have been elucidated: (1) Release of metal ions by extracellular barriers such as capsule, cell wall, and plasma membrane. (2) Extrusion of metal ions via efflux pumps or by diffusion. (3) Intracellular sequestration of metal ions. (4) Extracellular sequestration of metal ions. (5) Bio-transformation/detoxification of toxic metal ions. (6) Decreased sensitivity of cellular targets to metal ions. In general, heavy metal resistance-encoding genes are carried on mobile genetic elements such as plasmids and transposons or on chromosomal DNA (Rouch et al., 1995; Silver and Phung le, 2005; Ianeva, 2009; Seiler and Berendonk, 2012; Hobman and Crossman, 2014). In the following sections, we will briefly discuss these mechanisms.

#### Extrusion of Metal Ions via Efflux Pumps or Diffusion

Efflux pumps are the most prevalent bacterial tools conferring heavy metals resistance. This is achieved via Adenosine triphosphate (ATP) hydrolysis and/or through an electrochemical gradient of protons (Ianeva, 2009). Five major efflux system families are present in microorganisms: (1) ATP Binding Cassettes (ABC) family. (2) Resistance, Nodulation, Cell Division (RND) family. (3) Small Multi-Drug Resistance (SMR) family. (4) Multi-Drug and Toxic Compounds Efflux (MATE) transporters. (5) Major Facilitator Superfamily (MFS) family. These pumps differ in their amino acid sequence, substrate specificity, and energy consumption in pumping metal ions. For example, ATP Binding Cassettes (ABC) play a role in the efflux of metal ions and antimicrobial agents driven by ATP hydrolysis, while RNDs and SMRs pump out metal cations and antimicrobial agents via Chemiosmosis/Proton motive force (Silver and Phung le, 2005; Kourtesi et al., 2013; Abbas et al., 2017). Basal levels of efflux are usually not sufficient to confer heavy metal resistance in most bacterial species. However, changes in expression, either through mutations in promoter regions or efflux pump regulators, or inactivation of repressors, can lead to the over-expression of an efflux pump or confer resistance (Li et al., 2015; Blanco et al., 2016).

#### Intracellular Sequestration of Metal Ions

One important resistance mechanism involves intracellular metal ions sequestration upon binding to metal ions binding proteins [Metallothioneins (MTs), Glutathione (GSH), and Metallochaperones] (Ianeva, 2009). For example, Ni can complex with PO^3–^_4_, leading to its intracellular precipitation. *Staphylococcus aureus*, *Providencia* spp., *Vibrio harveyi*, *Shewanella* spp., and *Bacillus* spp., can precipitate Pb as phosphate salts (Levinson et al., 1996; Smeaton et al., 2009; Shin et al., 2012). Cd, Hg, Ag, Pb, and Zn can be trapped on cysteine-rich MT polypeptides that provide tolerance to high concentrations of heavy metals. For example, *Synechococcus* spp., *Pseudomonas* spp., and *Anabaena* spp. tolerate high concentrations of heavy metals via MT trapping mechanisms (Olafson et al., 1988; Naik et al., 2012). Some bacteria use GSH as an alternative chelator to sequester metal ions. GSH scavenges and detoxifies metals via its thiol (R-SH) group. Lima et al. (2005), demonstrated the role of GSH in mediating tolerance to Cd in *Rhizobium leguminosaru*. Finally, metallochaperones like Cu chaperones (Cu^1+^ binding chaperone CusF, Cu^1+^, and Cu^2+^ periplasmic chaperones PcoC and PcoE) can bind, trap, and transport metal ions to metalloenzymes and thus, decrease their toxic effects and protect cellular compartments (Yang et al., 2010; Pal et al., 2017).

#### Extracellular Sequestration of Metal Ions

In addition to intracellular sequestration, extracellular sequestration of heavy metals is an additional mechanism conferring bacterial resistance. This strategy provides a “Pre-defense” strategy as it occurs outside the bacterial cell. It involves the secretion of extracellular chelating proteins such as siderophores, oxalateoxalate, phosphate, and sulfide. However, this mechanism is mainly active in static environments when constant concentrations of heavy metals are present (Cunningham et al., 1993; Rouch et al., 1995). For example, *Streptomyces acidiscabies* can sequester Ni via hydroxamate siderophores while *Clostridium thermoaceticum* use sulfide to sequester Cd (Cunningham et al., 1993; Dimkpa et al., 2008).

#### Bio-Transformation/Detoxification of Toxic Metal Ions

Enzymatic detoxification reduces metal toxicity, which is accomplished via oxidation, reduction, and methylation reactions (Rouch et al., 1995; Silver and Phung le, 2005). For example, Hg^2+^ is reduced to a less toxic Hg^0^ form via Mercury(II) reductase encoded by *merA* gene (Seiler and Berendonk, 2012; Pal et al., 2017). In addition, upon bacterial entry Cr^6+^ is reduced to Cr^3+^ while As^3+^ is oxidized to As^5+^ thus, decreasing their toxicity (Silver and Phung le, 2005). Interestingly, *Pseudomonas* spp., and *Acinetobacter* spp., induce Pb methylation to minimize its toxic effects. To date, Hg methylation has only been only documented in anaerobic bacteria (Parks et al., 2013; Jaroslawiecka and Piotrowska-Seget, 2014).

#### Decreased Sensitivity of Cellular Targets to Metal Ions

Reducing sensitivity of cellular targets to metal ions is a way to minimize heavy metals toxicity. This is fulfilled via several mechanisms: (1) Decreasing bacterial susceptibility to metals by introducing mutations in resistance genes or determinants, or increasing the expression of the metal target site. (2) Producing a more resistant form of the metal target site upon activating an alternative target encoded on a plasmid. (3) Repairing DNA damage upon the activation of an SOS response which is the case of Cr-induced DNA lesions (Frohlich, 2013; Maret, 2015).

### Heavy Metals in Weapons

Explosives harbor huge amounts of Pb and Hg [Mercury(II) fulminate] (Navy U. S., 2008; Gebka et al., 2016). Zn, Cu, Ni, Pb, and Cr are used to coat bullets, missiles, gun barrels, and military vehicles (Audino, 2006; Casey, 2009). Ba, Sb, and B are weapon priming compounds, (Fitchett, 2019) and W is a kinetic bombardment due to its high density (19.3 g/cm^3^) (Rowlatt, 2014). In general, the use of heavy metals in weapons has increased since the end of World War II (Gebka et al., 2016).

### Known Resistance Mechanisms to Heavy Metals Frequently Found in Ordnance

#### Copper (Cu)

Copper (Cu) exists in nature as a free metallic element or alternates between 2 oxidative states Cu^1+^ and Cu^2+^. In humans, Cu is essential for blood vessel elasticity, brain development, maintenance of immune responses, and neurotransmitter production (Dorsey et al., 2004; Copper Development Association, 2018). Excess concentrations of Cu lead to kidney and liver damage, neurological, and immune diseases (Dorsey et al., 2004). In wars, Cu, Ni, Pb, and Cr are heavily used as coatings for bullets, missiles, gun barrels, and in military vehicles (tanks, trucks, and aircrafts). This could increase exposure to Cu in wartime and might explain increased observation of *A. baumannii* in studies conducted in these settings (Davis et al., 2005; Calhoun et al., 2008; Williams et al., 2016). Extensive studies on *Escherichia coli* and *Pseudomonas* spp. reveal four Cu homeostatic resistance systems: Cue, Cus, Pco, and Cop. Cue and Cus are chromosomally encoded efflux systems while Pco and Cop are plasmid encoded resistance systems (Pal et al., 2017).

#### Cue System

**FIGURE 1:**
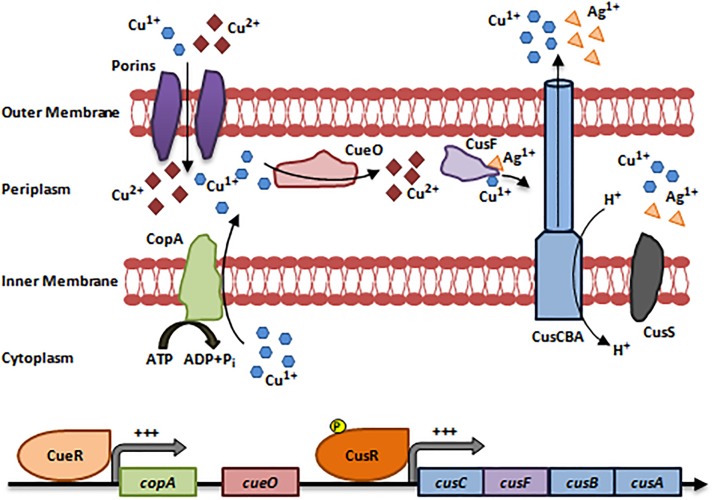
Resistance to Copper via the Cue and Cus systems. The Cue system is activated at low Cu concentrations and under aerobic conditions. Cu^1+^/Cu^2+^ enter the bacterial cells via non-specific porin proteins. CueR senses an increase in intracellular Cu concentrations and activates the expression of *copA* and *cueO*. Then, CopA translocate Cu^1+^ ions into the periplasm thus, protecting Cu-sensitive cytoplasmic compartments. In the periplasm, CueO oxidizes Cu^1+^ ions to the less toxic form Cu^2+^. The Cus efflux system is activated at high Cu concentrations, is strictly anaerobic, and pumps out Cu and Ag ions. Cu^1+^/Ag^1+^ ions enter the periplasm and induce the activation of CusS, which in turn phosphorylates and activates CusR. CusR induces the expression of *cusCFBA* operon. The protein products CusC, CusB, and CusA form a multi-Cu/Ag efflux pump (CusCBA) that pumps out Cu^1+^/Ag^1+^ ions after being transferred by the CusF metallochaperone (Franke et al., 2003; Delmar et al., 2015; Pal et al., 2017). Adapted and modified with permission from Pal et al. (2017).

The Cue system (Copper Efflux) is active at low Cu concentrations and under aerobic conditions. It consists of an inner membranous Cu^1+^ exporting P-type ATPase (CopA) and a periplasmic multi-Cu Oxidase (CueO). *copA* and *cueO* are activated by a cytoplasmic transcriptional Cu-responsive Regulon (CueR) upon sensing increased Cu concentrations (Hobman and Crossman, 2014; Delmar et al., 2015; Pal et al., 2017; Figure 1).

#### Cus System

Unlike the Cue system, the Cus efflux system is active at high Cu concentrations, is strictly anaerobic, and pumps out Cu and Ag cations (Silver and Phung le, 2005; Singh S.K. et al., 2011; Delmar et al., 2015). It detoxifies Cu in the periplasmic compartment, unlike the Cue system that extrudes periplasmic and cytoplasmic Cu (Jaroslawiecka and Piotrowska-Seget, 2014). The Cus system consists of 4 genes forming the *cusCFBA* operon, which is regulated by a two-component regulatory system (CusS/CusR) (Franke et al., 2003; Silver and Phung le, 2005; Pal et al., 2017). CusS, a histidine kinase, is activated upon Cu/Ag stimulation, while CusR is a DNA-binding transcriptional activator that activates *cusCFBA* expression. CusC, CusB, and CusA form a multi-Cu/Ag efflux pump (CusCBA) that functions as a proton-ion antiporter. Cu^1+^/Ag^1+^ are transported to CusCBA via the periplasmic metallo-chaperone, CusF (Franke et al., 2003; Silver and Phung le, 2005; Pal et al., 2017). Cu resistance via the Cue and Cus systems are detailed in Figure 1.

#### Pco System

**FIGURE 2:**
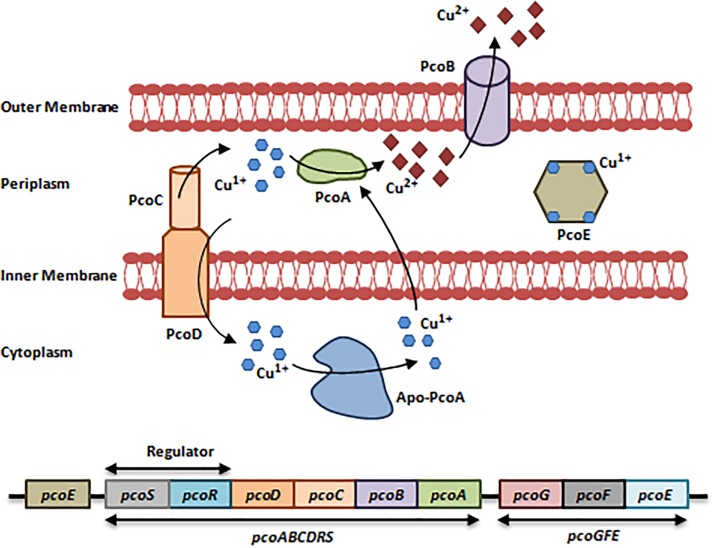
Resistance to Copper via the Pco system. The Pco system consists of 2 operons, *pcoGFE* and *pcoABCDRS* in addition to a single gene *pcoE*. This system cannot function independently; it requires the activity of the Cue system and CopA in specific to induce resistance to Cu, which is heavily present in bullets, missiles, gun barrels, and in military vehicles. First, Cu^1+^/Cu^2+^ enter the bacterial cell via non-specific porin proteins. PcoD transports Cu^1+^ into the cytoplasm. Cu^1+^ is toxic in the cytoplasm; Apo-PcoA transports Cu^1+^ back to the periplasm and PcoR/PcoS senses an increase in Cu concentrations and in turn induces the expression of *pcoGFE* and *pcoABCDRS*. In addition to Apo-PcoA, the periplasmic Cu-chaperone PcoC transports Cu^1+^ to the periplasm, where CopA from the Cue system and PcoA oxidize Cu^1+^ to the less toxic form Cu^2+^. Cu^2+^ ions are expelled out via the PcoB efflux pump. Finally, PcoE is a metallochaperone, which is believed to provide initial bacterial resistance to Cu upon its entry through sequestering Cu^1+^ ions until the activation of the Pco system is fulfilled (Bondarczuk and Piotrowska-Seget, 2013; Pal et al., 2017). Adapted and modified with permission from Pal et al. (2017).

The *E. coli*-resistant Pco system (Plasmid-borne-Copper Resistance) found in Cu-fed pigs consists of two operons, *pcoGFE*, and *pcoABCDRS* that are encoded by a 9-10 gene cluster (Brown et al., 1995). Like the Cus system, the Pco system is regulated by a two-component regulatory system (PcoR/PcoS) (Brown et al., 1995; Pal et al., 2017). To actively function, the Pco system requires the action of CopA from the Cue system in addition to PcoA and PcoC. PcoA is a multi-Cu Oxidase that oxidizes Cu^1+^ to Cu^2+^, while PcoC is a periplasmic Cu-binding protein that acts as a chaperone which delivers Cu^1+^ to CopA during oxidation and to PcoD. PcoD is an inner membrane Cu transporter that is involved in Cu uptake. PcoB and PcoE are an outer membrane transporter and a metallo-chaperone, respectively (Figure 2; Lee et al., 2002; Bondarczuk and Piotrowska-Seget, 2013; Pal et al., 2017).

#### Cop System

The Cop system is encoded by a cluster of 6 plasmid-borne genes arranged in two operons, *copABCD* and *copRS* (Pal et al., 2017). *copABCD* and *copRS* are homologs of *pcoABCDRS*. The Cop determinants are genetically associated with the Cop system and have similar roles. *copABCD* is under the regulation of CopR/CopS. Protein products are associated with Cu sequestration in the periplasm and outer membrane (Bondarczuk and Piotrowska-Seget, 2013; Pal et al., 2017). In *Cupriavidus metallidurans* CH34 and *E. coli*, Cu ions can be sequestered by CusF in the periplasm, exported by the RND-driven CusCBA efflux pump, or oxidized to Cu^2+^ (Nies, 2016).

#### Mercury (Hg)

Mercury (Hg) is released into the environment via geological and human activities such as soil and rock erosion, volcanic eruptions, mining, and fuel combustion (Tchounwou et al., 2012). In the wake of recent conflicts in Lebanon, Syria, Iraq, Yemen, and Afghanistan, the Middle East has become one of the most polluted regions with Hg (Gworek et al., 2017). This may have led to an increased bacterial tolerance to this metal. In humans, Hg has no biological role and at very low concentrations it is fatal, leading to brain, lung, and kidney failure (Risher and Rob, 1999).

**FIGURE 3:**
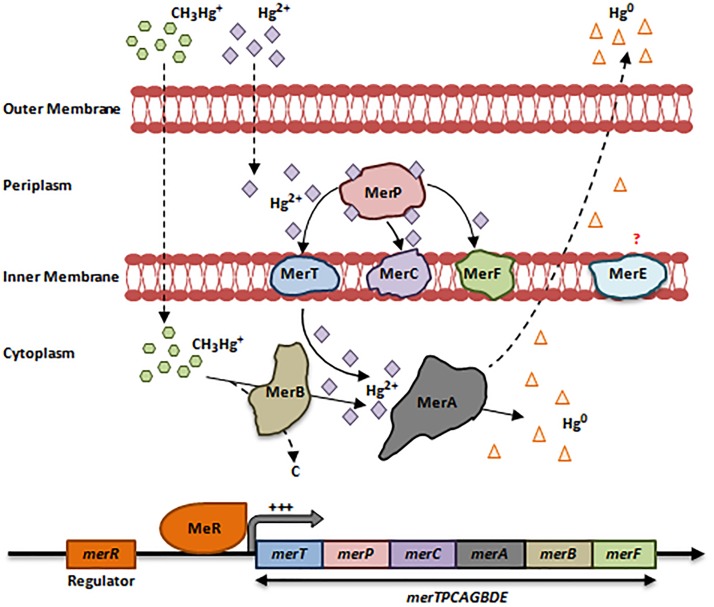
Bacterial resistance to mercury. The *mer* operon consists of a cluster up to 8 genes *merTPCAGBDE*. Upon the entry of the inorganic form of Hg (Hg^2+^) via non-specific porin proteins, the first protein to bind it is MerP, a small periplasmic chaperone. MerP transports Hg^2+^ to MerT, MerC or MerF, which are inner membranous mercuric ions-binding proteins that in turn transport Hg^2+^ to the cytoplasm. *merT* is the most prevalent gene within the *mer* operon as compared to *merC* and *merF*. In the cytoplasm, MerA detoxifies Hg^2+^ ions through reduction-catalyzed volatilization process to a non-toxic elemental form Hg^0^. This form is volatile at room temperature; it diffuses outside the membranes allowing the bacterial cell to escape Hg toxicity (Nies, 1999; Silver and Phung le, 2005; Boyd and Barkay, 2012; Hobman and Crossman, 2014). MerE is an inner membranous protein of unknown function (Silver and Phung le, 2005). In Gram-negative bacteria, the *mer* operon is regulated by MerR, which is in turn activated by increased Hg^2+^ levels in the cytoplasm. This induces the expression of the whole *merTPCAGBDE* operon (Boyd and Barkay, 2012; Hobman and Crossman, 2014). Resistance to the organic form of Hg (CH_3_-Hg^+^) is achieved by *merB*, which encodes an Organomercurcial Lyase (MerB) located in the cytoplasm. When CH_3_-Hg^+^ enter the cytoplasm via non-specific porin proteins, MerB cleaves the Mercury-Carbon bond and releases Hg^2+^ in the cytoplasm. At this point, Hg^2+^ is reduced to Hg^0^ that diffuse outside the bacterial cell (Nies, 1999; Boyd and Barkay, 2012; Hobman and Crossman, 2014; Pal et al., 2017). Adapted and modified with permission from Silver and Phung le (2005), Boyd and Barkay (2012) and Pal et al. (2017).

Hg can access bacteria in 2 forms: organic (CH_3_-Hg^+^) and inorganic (Hg^2+^), both of which are toxic (Hobman and Crossman, 2014). Despite this toxicity, several bacterial species have developed resistance mechanisms to CH_3_-Hg^+^/Hg^2+^ via the *mer* operon, and are found mainly in war zone regions (Mirzaei et al., 2008; Pérez-Valdespino et al., 2013; Hobman and Crossman, 2014). The *mer* operon is present on plasmids and transposons and consists of a cluster of 8 genes *merTPCAGBDE* regulated by MerR (Silver and Phung le, 2005; Hobman and Crossman, 2014). This operon encodes a chain of proteins that bind CH_3_-Hg^+^/Hg^2+^ and oxidize them, such as MerA [Mercury(II) Reductase], the key player in Hg^2+^ detoxification (Figure 3).

#### Arsenic (As)

Arsenic (As) is released into the environment from soil and rock erosion, volcanic eruptions, mining, and crops treated with pesticides and herbicides (Paez-Espino et al., 2009; Tchounwou et al., 2012). In 1918, two organic As compounds, Lewisite (C_2_H_2_AsCl_3_) and Adamsite (C_12_H_9_AsClN) were developed by the US army as chemical weapons; both are classified as potential bioterrorism agents by CDC (2013). Agent Blue, an arsenical mixture of cacodylic acid and sodium cacodylate was sprayed by the United States on crops as part of “resource deprivation” strategies in the Vietnam war beginning in 1962 (Radke et al., 2014). The use of chemical weapons in the Syrian Civil War has been confirmed by the United Nations. This resulted in increased bacterial resistance to As via oxidation, reduction, methylation, efflux, and intracellular sequestration on cysteine-rich peptides (Silver and Phung le, 2005; Paez-Espino et al., 2009), and was associated with detrimental health effects ranging from cardiovascular disease, respiratory disorders, gastro-intestinal symptoms, hematological disorders, diabetes, neurological, and developmental anomalies (Chou et al., 2007; Tchounwou et al., 2012).

**FIGURE 4:**
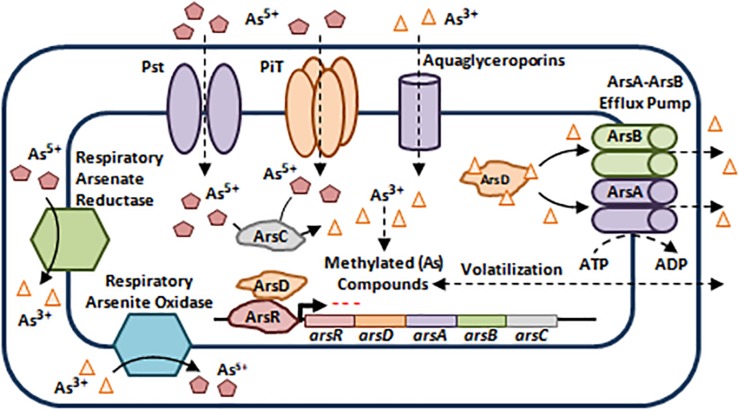
Bacterial resistance to arsenic. (As) enter the bacterial cell using Phosphate-Specific Transporters (Pst) and Type III Transporters (PiT) in the case of As^5+^ and Aquaglycerolporins in the case of As^3+^ (Paez-Espino et al., 2009). The *ars* operon harbors 3 co-transcribed core genes that confer resistance not only to As^3+^ and As^5+^, but also to Antimony (Sb^3+^). *arsR* encodes a Transcriptional Repressor, *arsC* encodes a Cytoplasmic Arsenate Reductase, and *arsB* encodes a membrane bound Arsenite Efflux Pump. Two additional genes may be present within the *ars* operon, *arsA* and *arsD*. The former encodes an intracellular ATPase which binds ArsB to form an ArsA-ArsB ATPase Efflux Pump, while the latter is a metallochaperone that binds and delivers As^3+^ and Sb^3+^ to ArsA-ArsB complex for efflux, in addition to its role as a trans-activating co-repressor of the *ars* operon along with ArsR (Silver and Phung le, 2005; Paez-Espino et al., 2009; Hobman and Crossman, 2014). Moreover, some microorganisms escape As toxicity by methylation thus, leading to the production of less toxic and volatile derivatives that diffuse outside the bacterial cell (Paez-Espino et al., 2009). Besides As toxicity, bacteria belonging to the *Shewanella* spp., *Sulfurospirillum* spp., *Clostridium* spp., and *Bacillus* spp., use As^5+^ as a final electron acceptor during anaerobic respiration by reducing it to As^3+^, while other bacteria use As^3+^ as an electron donor and oxidize it to As^5+^ during aerobic oxidation (Paez-Espino et al., 2009). The oxidation/reduction processes are mediated by the Respiratory Arsenate Reductase and Respiratory Arsenite Oxidase that are encoded by the *arrAB* operon and *asoAB* genes respectively (Silver and Phung le, 2005; Paez-Espino et al., 2009). Adapted and modified with permission from Paez-Espino et al. (2009).

As exists in two chemical forms: inorganic and organic. Inorganic As occurs as pentavalent Arsenate (As^5+^), trivalent Arsenite (As^3+^), elemental Arsenic (As^0^), and Arsenide (As^3–^) with As^3+^ and As^5+^ being the most toxic inorganic forms and most prevalent in nature (Nies, 1999; Paez-Espino et al., 2009; Tchounwou et al., 2012). Organic As is less toxic than inorganic arsenicals (Chou et al., 2007). Bacterial resistance to As is mainly encoded by efflux via the *ars* operon, which can be plasmid or chromosomally driven, even though it can also be encoded by other genetic determinants such as *arr* genes and *aox* genes (Figure 4; Silver and Phung le, 2005; Paez-Espino et al., 2009).

#### Chromium (Cr)

Chromium (Cr) is the 7th most abundant heavy metal in the earth’s crust and is present in nature in several oxidation states ranging from divalent (+2) to hexavalent (+6) Cr^3+^ and Cr^6+^ are the most stable. While Cr^3+^ is naturally present in the environment, Cr^6+^ is mostly produced by industrial processes such as mining, electroplating, dye production, and leather tanning (Ahemad, 2014; Joutey et al., 2015; Pradhan et al., 2016). In weapons, Cr was initially used by the Chinese to coat metal weapons (Dunham, 2019). Nowadays, Cr is heavily used to coat gun barrels, where it is used as a bore protection (Audino, 2006). Moreover, Cr levels were highest in deciduous teeth from Iraqi patients during the Iraqi war, which highlights the heavily polluted Middle Eastern region with heavy metals (Savabieasfahani et al., 2016). The solubility and oxidizing potential of Cr^6+^ makes it 1000× more toxic to humans as compared to Cr^3+^, and this makes it a strong factor associated with nasal and bronchogenic carcinomas (International Agency for Research on Cancer [IARC], 1990; Ahemad, 2014).

**FIGURE 5:**
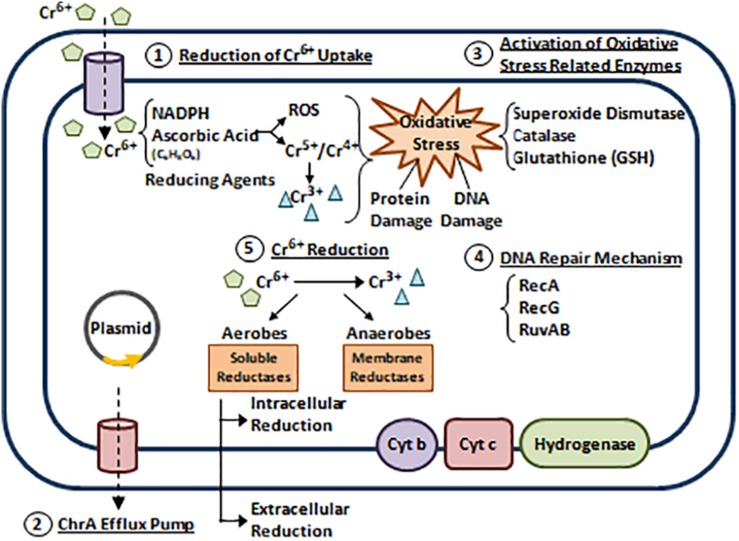
Bacterial resistance to chromium. Five Cr resistance mechanisms are reported (Camargo et al., 2005; Joutey et al., 2015). (1) Reduction of Cr^6+^ uptake. Cr^6+^ exists in the form of Oxyanions Chromate (CrO_4_^2–^) and Dichromate (Cr_2_O_7_^2–^). Bacterial cells can reduce Cr^6+^ uptake via the sulfate transport system (Nies, 1999; Ahemad, 2014; Joutey et al., 2015). (2) Cr^6+^ efflux. Studies reveal that *P. aeruginosa* and *Alcaligenes eutrophus* can extrude Cr^6+^ by active efflux through ChrA (Chromate) pump (Collard et al., 1994; Alvarez et al., 1999). In 2008, a plasmid encoded operon (*chrBACF*) was identified in *Ochrobactrum tritici* responsible for Cr efflux, where *chrB* and *chrA* are the main genes involved (Branco et al., 2008). (3) Activation of oxidative stress related enzymes. When Cr^6+^ enter the bacterial cell, it interacts with reducing agents such as Nicotinamide Adenine Dinucleotide Phosphate (NADPH) and Ascorbic Acid to produce free radicals and unstable Cr intermediates (Cr^4+^ and Cr^5+^) that are further reduced to Cr^3+^. End products of these reactions cause oxidative stress leading to protein and DNA damage. This induces the up-regulation of antioxidants enzymes that scavenge ROS and protect cellular compartments (Ahemad, 2014; Joutey et al., 2015; Pradhan et al., 2016). (4) Repairing DNA damage induced by Cr^6+^ and its derivatives. This is achieved via SOS response activation. Several studies highlight the roles of RuvAB, RecA, and RecG (helicases) in mediating Cr resistance through repairing Cr^6+^ induced DNA damage (Miranda et al., 2005; Morais et al., 2011). (5) Cr^6+^ reduction. Cr^6+^ can be reduced aerobically or anaerobically to a less toxic form Cr^3+^. Aerobic reduction uses cytoplasmic soluble reductases and NADPH, while anaerobic reduction uses membrane reductases belonging to the electron transport chain (cytochromes b and c, and hydrogenases) (Morais et al., 2011; Ahemad, 2014; Joutey et al., 2015). Adapted and modified with permission from Ahemad (2014).

In bacteria, Cr has no metabolic role and thus, it is toxic in several species such as *Pantoea* spp., *Aeromonas* spp., *Acinetobacter* spp., and *E. coli* (Nies, 1999). However, many bacteria developed Cr resistance via 5 reported mechanisms that are mostly plasmid encoded (Camargo et al., 2005; Joutey et al., 2015). (1) Reduction of Cr^6+^ uptake. (2) Cr^6+^ efflux. (3) Activation of oxidative stress related enzymes. (4) Repairing DNA damage induced by Cr^6+^ and its derivatives. (5) Cr^6+^ reduction (Figure 5; Branco et al., 2008; Panda and Sarkar, 2012; Ahemad, 2014; Joutey et al., 2015; Pradhan et al., 2016).

#### Lead (Pb)

Lead (Pb) is predominantly released into the environment from human activities such as manufacturing pipes, X-ray shields, lead-acid storage batteries, munitions, and bullets (Abadin et al., 2007; Jaroslawiecka and Piotrowska-Seget, 2014). It exists in two main oxidative states (Pb^2+^ and Pb^4+^). In addition to bullets, Pb is present in explosives that ignite gunpowder. It usually vaporizes upon firing and thus, Pb fumes and dust are inhaled, leading to brain damage, anemia, and high blood pressure (Abadin et al., 2007; Dermatas and Chrysochoou, 2007).

**FIGURE 6:**
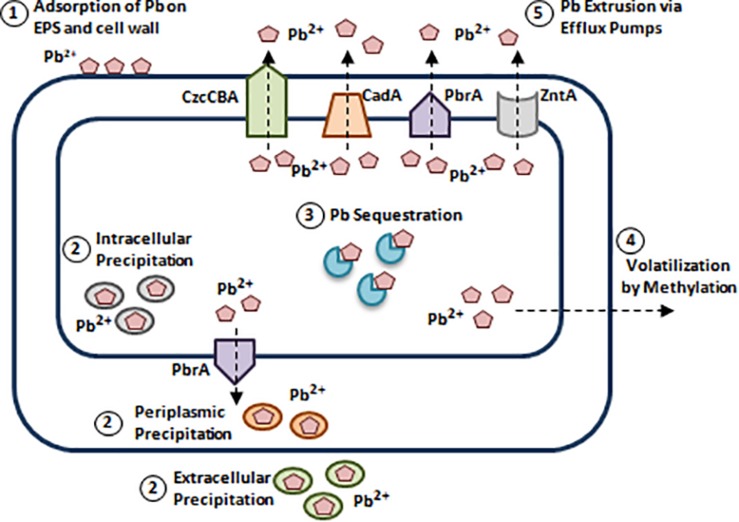
Bacterial resistance to lead. Bacterial species such as *Pseudomonas* spp., and *Acinetobacter* spp., developed Pb resistance mechanisms. (1) Adsorption of Pb on EPS and bacterial cell wall. Structures like cell wall and extracellular polymers can adsorb Pb^2+^ due to the presence of negatively charged functional groups [Carboxyl (C(= O)OH), Hydroxyl (R-OH), and Phosphate groups (PO^3–^_4_)] (Jaroslawiecka and Piotrowska-Seget, 2014). (2) Reducing Pb accumulation via intracellular and extracellular precipitation. *S. aureus*, *Providencia* spp., and *Pseudomonas* spp., can precipitate Pb intracellularly in the form of Lead(II) phosphate [Pb_3_(PO_4_)_2_], while in *Citrobacter freundii*, extracellular Pb precipitation is mediated by phosphatase. In addition to intracellular and extracellular precipitation, periplasmic precipitation of Pb involves adsorption to polymers present in the cell wall (al-Aoukaty et al., 1991; Levinson et al., 1996; Jaroslawiecka and Piotrowska-Seget, 2014). (3) Pb sequestration via intracellular proteins. Pb binding-MTs were reported in Pb resistant *P. aeruginosa* strain WI-1 and *Providencia vermicola* strain SJ2A. This is mediated by a plasmid-borne MT encoding gene, *bmtA* responsible for Pb sequestration (Naik et al., 2012; Sharma et al., 2017). (4) Pb detoxification via methylation. Methylation of Pb is documented in *Acinetobacter* spp., *Pseudomonas* spp., *Aeromonas* spp., and others. Arctic marine bacteria convert inorganic Pb to tri-methyl-lead (C_3_H_9_Pb), while *Acinetobacter* spp., convert it to tetra-methyl derivatives (Wong et al., 1975; Jaroslawiecka and Piotrowska-Seget, 2014). (5) Pb extrusion via efflux pumps. Pb efflux is mediated by P-type ATPases such as, CadA of *S. aureus*, ZntA of *E. coli*, and PbrA of *Cupriavidus metallidurans* and to a lower extent by RND/CBA chemiosmotic transporters. CadA, ZntA, and PbrA are homologous P-type ATPases that can pump out Pb^2+^, Zn^2+^, and Cd^2+^ (Leedjärv et al., 2007; Jaroslawiecka and Piotrowska-Seget, 2014). Adapted and modified with permission from Jaroslawiecka and Piotrowska-Seget (2014).

Pb toxicity involves inducing cellular damage through ROS formation, disrupting enzymatic conformations, and interfering in calcium (Ca) metabolism (Tchounwou et al., 2012). Due to the widespread Pb contamination, bacteria have developed Pb resistance mechanisms (Levinson et al., 1996; Jaroslawiecka and Piotrowska-Seget, 2014). (1) Adsorption of Pb on EPS and bacterial cell wall. (2) Reducing Pb accumulation via intracellular and extracellular precipitation. (3) Pb sequestration via intracellular proteins. (4) Pb detoxification via methylation. (5) Pb extrusion via efflux pumps (Figure 6; Levinson et al., 1996; Silver and Phung le, 2005; Jaroslawiecka and Piotrowska-Seget, 2014; Subramanian, 2018).

### Association Between Heavy Metals and AMR

Worldwide concerns about heavy metal contamination, resistance, and its ability to induce AMR are increasing. These concerns are associated with the heavy metals used in manufacturing weapons; most heavy metals are non-biodegradable and persist in the environment. Moreover, many bacterial species evolved resistance mechanisms to combat metals toxicity (Seiler and Berendonk, 2012; Yu et al., 2017). These mechanisms are encoded by resistant genes to heavy metals and antimicrobial agents that are physically linked on mobile genetic elements (Seiler and Berendonk, 2012; Yu et al., 2017). More importantly, heavy metals can induce selective pressure on microbial populations leading to antimicrobial resistance through a mechanism called “co-selection” which occurs via 3 major ways (Seiler and Berendonk, 2012).

### Co-resistance

Co-resistance occurs when genes encoding resistance to heavy metals and antimicrobial agents are physically linked/located in close proximity to each other on mobile genetic elements such as plasmids, genomic islands (GIs), transposons, or integrons (Trevors and Oddie, 1986; Seiler and Berendonk, 2012; Yu et al., 2017). For example, in Cu-resistant *Enterococcus faecium* isolated from pigs, *tcrB* and genes encoding resistance to erythromycin and vancomycin are encoded on the same conjugative plasmid (Hasman and Aarestrup, 2002; Silveira et al., 2014). Moreover, in *Serratia marcescens*, plasmid-borne resistance to chloramphenicol, kanamycin, and tetracycline is genetically linked to As, Cu, Hg, and Ag resistance genes (Gilmour et al., 2004). Interestingly, Whole-Genome Sequencing (WGS) analysis in *Salmonella typhi* reveal a genetic association between Hg resistance and several unrelated antimicrobial agents resistance genes (chloramphenicol, ampicillin, streptomycin, sulfonamide, and trimethoprim) (Wireman et al., 1997).

### Cross-Resistance

Cross-resistance occurs when one resistance mechanism confers resistance to heavy metals and antimicrobial agents simultaneously. This is mainly achieved via multi-drug efflux pumps (Baker-Austin et al., 2006; Pal et al., 2017). The MdrL efflux pump in *Listeria monocytogenes* encodes resistance to Zn, Co, Cr, erythromycin, josamycin, and clindamycin (Mata et al., 2000). Moreover, the DsbA-DsbB (Disulfide Bond) multi-drug efflux system in *Burkholderia cepacia* induces cross-resistance to β-lactams, kanamycin, erythromycin, novobiocin, ofloxacin, and Zn^2+^ and Cd^2+^ metal ions (Hayashi et al., 2000). In addition, resistance to antimicrobial agents, Co, and Cu is mediated via the CmeABC multi-drug efflux pump in *Campylobacter jejuni* (Lin et al., 2002).

### Co-regulatory Resistance

Co-regulation is the least common mechanism of co-selection. It is fulfilled when resistant genes to antimicrobial agents and heavy metals are controlled by a mutual regulatory protein (Pal et al., 2017). A very well characterized co-regulatory resistance system is the CzcS-CzcR two component regulatory system of *P. aeruginosa*. This system induces resistance to Zn^2+^, Cd^2+^, and Co^2+^ by activating the expression of *czc*CBA (Cobalt Zinc Cadmium) efflux pump and to the carbapenem imipenem by suppressing expression of the OprD porin encoding gene (Perron et al., 2004).

### Whole-Genome Sequencing and Heavy Metal Resistance

Many reports highlighted the importance of WGS as an effective tool to detect genome-wide modifications and the emergence of heavy metal resistance genes. Nowadays, it is feasible to assess the entire bacterial genome at low costs and in a timely manner, making it an ideal method for AMR surveillance. Therefore, WGS provides a practical solution to evaluate genomes and determine resistant genes for compounds that are not frequently assessed. Moreover, this tool allows scientists to discover novel resistance mechanisms and provides valuable information to researchers and clinicians in antimicrobial prescriptions (Khromykh and Solomon, 2015; Jagadeesan et al., 2019). For example, the arsenic resistance cassette, *arsRCDAB*, present on a class 1 integron and mobilized on a conjugative plasmid was detected in two *Salmonella enterica* isolates from Singapore with high tolerance to arsenate (Wilson et al., 2019). Moreover, heavy metal resistant genes to Manganese (Mn^2+^) and Cd^2+^ in addition to exopolysaccharides production (EPS) were documented upon WGS analysis in *Pseudaminobacter manganicus* isolated from a manganese mine. This aspect sheds light on metal removal/adsorption and reflects bioremediation capabilities in contaminated regions (Xia et al., 2018). In *A. baumannii*, WGS analysis revealed the role of two integrases in the excision and circularization of heavy metal resistance (GIs) in *E. coli* (Al-Jabri et al., 2018). Bacterial WGS and typing databases such as BacWGSTdb (PMID: 26433226)^1^ serve as easy, rapid, powerful, and convenient tools to assess AMR and provide valuable information to WGS analysis and daily use in the clinical microbiology laboratories (Ruan and Feng, 2016). Moreover, “BacMet: Antibacterial Biocide and Metal Resistance Genes Database” provides an accurate and high quality resource of bacterial genes associated with heavy metal resistance present in literature^2^. These available databases serve as important tools to help understand bacterial resistance mechanisms to heavy metals by linking various factors and parameters involved.

## Conclusion

The reason for the rapid emergence of drug resistant *A. baumannii* in war-wounded patients remains unclear. Heavy metal contaminated areas may be driving the increase in antimicrobial resistance. This may explain an observed increase in bacterial resistance to both heavy metals and antimicrobial agents and lead to the development of novel mechanisms of resistance. The role of this pathway in *A. baumannii* is poorly understood.

Until now antimicrobial resistance has been largely attributed to poor antimicrobial stewardship in humans and in animals. The mechanisms described above, whereby heavy metals may produce antimicrobial resistance in their absence, identifies a potential pathway driving global antimicrobial resistance that would not be addressed through improved antimicrobial stewardship. This pathway would be facilitated in wartime, and could explain the emergence of previously little reported pathogens as possible amplification points along this pathway.

Further research into the role of heavy metals driving antimicrobial resistance, its influence in war zones, and the contribution of *A. baumannii* as a reservoir, are therefore warranted given the implications for addressing the global AMR crisis.

## Author Contributions

AGA, WB, AN, L-PH, MZ, and PH contributed to reviewing the literature and the write up of the manuscript. V-KN, HL, OD, GA-S, MM, NK, AA, CK, and GM contributed to the editing of the manuscript.

## Conflict of Interest

The authors declare that the research was conducted in the absence of any commercial or financial relationships that could be construed as a potential conflict of interest.

## Footnotes

1 http://bacdb.org/BacWGSTdb

2 http://bacmet.biomedicine.gu.se/
